# N6-Methylandenosine-Related lncRNAs in Tumor Microenvironment Are Potential Prognostic Biomarkers in Colon Cancer

**DOI:** 10.3389/fonc.2021.697949

**Published:** 2021-06-11

**Authors:** Hongliang Zhang, Lei Zhao, Songyan Li, Jing Wang, Cong Feng, Tanshi Li, Xiaohui Du

**Affiliations:** ^1^ Medical School of Chinese People's Liberation Army (PLA), Beijing, China; ^2^ Department of Emergency, The First Medical Center, Chinese PLA General Hospital, Beijing, China; ^3^ The 65651 Army of the Chinese PLA, Jinzhou, China; ^4^ Department of Clinical Laboratory, Peking University People’s Hospital, Peking University People’s Hospital, Beijing, China; ^5^ Department of General Surgery, The First Medical Center, Chinese PLA General Hospital, Beijing, China

**Keywords:** colon cancer, tumor microenvironment, N6-methylandenosine (m6A), lncRNA - long noncoding RNA, prognostic biomarker

## Abstract

**Background:**

LncRNA dysregulation and the tumor microenvironment (TME) have been shown to play a vital role in the progression and prognosis of colon cancer (CC). We aim to reveal the potential molecular mechanism from the perspective of lncRNA in the TME and provide the candidate biomarkers for CC prognosis.

**Methods:**

ESTIMATE analysis was used to divide the CC patients into high and low immune or stromal score groups. The expression array of lncRNA was re-annotated by Seqmap. Microenvironment-associated lncRNAs were filtered through differential analysis. The m6A-associated lncRNAs were screened by Pearson correlation analysis. Lasso Cox regression analyses were performed to construct the m6A- and tumor microenvironment-related lncRNA prognostic model (m6A-TME-LM). Survival analysis was used to assess the prognostic efficacy of candidate lncRNAs. Enrichment analyses annotated the candidate genes’ functions.

**Results:**

We obtained 25 common differentially expressed lncRNAs (DELs) associated with immune microenvironment and m6A-related genes for subsequent lasso analysis. Four out of these DELs were selected for the m6A-TME-LM. All the four lncRNAs were related to overall survival, and a test set testified the result. Further stratification analysis of the m6A-TME-LM retained its ability to predict OS for male and chemotherapy adjuvant patients and performed an excellent prognostic efficacy in the TNM stage III and IV subgroups. Network analysis also found the four lncRNAs mediated co-expression network was associated with tumor development.

**Conclusion:**

We constructed the m6A-TME-LM, which could provide a better prognostic prediction of CC.

## Introduction

Colon cancer (CC) is a refractory malignant tumor. The incidence of CC is increasing at an alarming rate due to the rapid industrialization of recent times and the changes in urban lifestyles worldwide. Almost 1.5 million people were diagnosed with colon cancer each year, and more than 500,000 deaths occurred every year. CC patients also accounted for 40% of cancer cases diagnosed each year (1). Treatment options for CC include targeted therapy, surgery, radiation therapy, chemotherapy, and cryosurgery (2). Chemotherapy is not effective because of its significant side effects and the risk of drug resistance in CC (3). Therefore, surgery is the primary treatment for colon cancer, and it can cure about half of the patients. However, the recurrence rate of colon cancer remains high after surgery, which is one of the causes of patients’ death (4). Although researchers have made considerable progress in early diagnosis and improving the efficiency of different treatments over time, the benefits of radiation and chemotherapy remain low. Thus, efficient prognostic markers are also of particular importance.

As a new treatment strategy, the treatment of tumor microenvironment (TME) has attracted public attention (5). TME is composed of a variety of cell types and plays a crucial role in the occurrence and progression of tumors (6). With the advances in tumor cytology and molecular biology, in-depth insight into TME is vital to reveal the fundamental molecular mechanisms and to improve immunotherapy (7, 8). The algorithm, Estimation of Stromal and Immune cells in MAlignant Tumor tissues using Expression data (ESTIMATE), could estimate the abundance of tumor-infiltrating immune cells based on gene expression (9, 10). Research shows that targeting stromal cells and connective tissue cells can be a new way to overcome drug resistance effectively (1).

Almost all mRNAs and lncRNAs undergo N6-methyladenosine (m6A) modification, an epigenetic methylation modification that plays a crucial role in RNA transport, translation, and other functions (11, 12). M6A modification is regulated by m6A regulators, such as methyltransferases (writers), signal transmitters (readers), and demethylases (erasers) (13). Many studies have shown that m6A modifications can be involved in the carcinogenesis and prognosis of various cancers, including colon cancer. For example, *ALKBH5* was downregulated in human colon cancer tissues and was significantly correlated with distant metastasis. A functional experiment found that overexpression of *ALKBH5* inhibited colon cancer cells invasion *in vitro* and metastasis *in vivo* (14); the m6A modification of the *PD-L1* mRNA and the binding of *FTO* to the *PD-L1* mRNA was testified in HCT-116 cell line by RNA immunoprecipitation assay which indicated that *FTO* could regulate *PD-L1* expression in colon cancer cells (15); and lncRNA *OCC-1* suppresses cell growth through destabilizing HuR (*ELAVL1*) protein in colorectal cancer (16); *METTL3* could maintain colon cancer tumorigenicity by suppressing *SOCS2* to promote cell proliferation (17).

Aberrant expression of lncRNA is also closely associated with malignant progression, and poor prognosis of tumors and lncRNA dysregulation has been shown to play a critical role in the development of colon cancer (18). For instance, *SOX9*-activated *FARSA-AS1* is reported to affect cell growth, stemness, and metastasis in colorectal cancer through upregulating *FARSA* and *SOX9* (19); *LOC441461* (*STX17-AS1*) could modulate colorectal cancer cell growth and motility (20); and *LINC01578* drives colon cancer metastasis through a positive feedback loop with the NF-κB/YY1 axis (21). However, the mechanism of aberrantly expressed lncRNAs regulated by m6A modification in colon cancer remains unclear, and few studies have investigated how m6A modification affects lncRNAs and thus participates in regulating colon carcinogenesis and progression. So, understanding how lncRNAs subject to m6A modification are involved in the malignant progression and poor prognosis of colon cancer can help researchers screen for biomarkers that can guide subsequent treatment.

**Figure 1:**
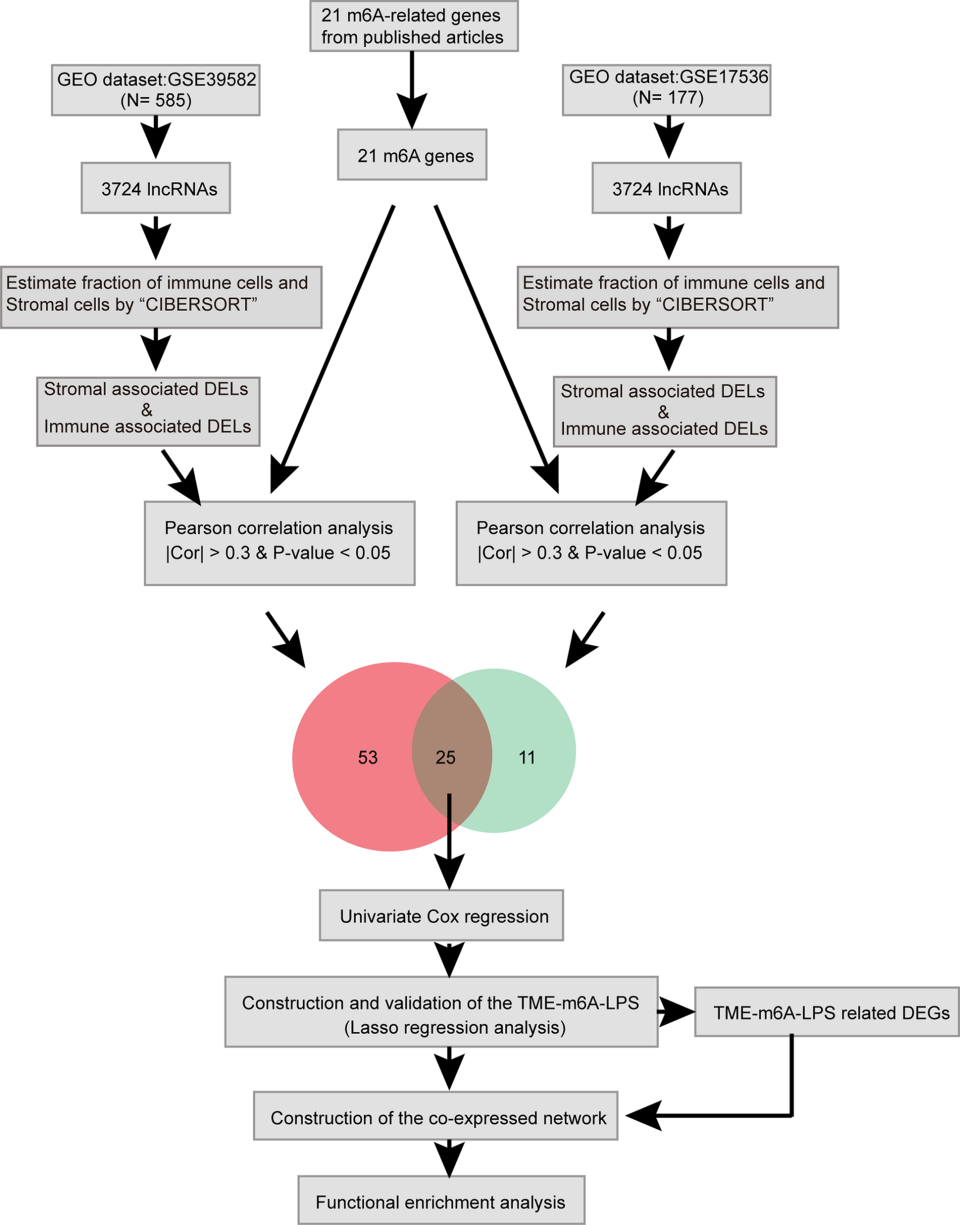
Study flow chart of the integration analysis.

Here, we identified lncRNAs which differentially expressed in TME and related to m6A modification by bioinformatic and statistical analysis and identified potential biomarkers which can predict CC prognosis **(** **Figure 1** **)**.

## Materials and Methods

### Data Sources

The expression data of 585 CC patients and corresponding clinical information were extracted from the GEO database with the accession number GSE39582. Another whole-genome gene expression data of 177 CC patients with matched clinical data were downloaded with the accession number GSE17536 (https://www.ncbi.nlm.nih.gov/geo/). Then, the lncRNA expression matrix of the TCGA CC data set was used as an independent test data set and downloaded from the TANRIC database (22). All the CC patients with complete overall survival (OS) information were selected. The gene expression profiles were quantified by FPKM and normalized through log2-based transformation. In addition, the ESTIMATE algorithm was employed to calculate immune and stromal scores for each sample by “estimate” R package.

### Re-Annotating Microarray to Construct lncRNA Expression Profiles

Annotation files for lncRNAs and protein-coding genes (PCGs) were downloaded from GENECODE (https://www.gencodegenes.org/human/). Moreover, the human genome annotation file (GRCh38/hg38) was downloaded from the UCSC database (http://hgdownload.cse.ucsc.edu/).

We mapped the probe sequences to the human genome (hg38) by Seqmap software (http://www-personal.umich.edu/~jianghui/seqmap/). First, the sequence of 54675 probes (HG-U133_Plus_2) was mapped to GRCh38 human reference genome by Seqmap, and the corresponding genome position of these probes was found. Then, remove probes that match to multiple locations, leaving probes that uniquely mapped to a single location in human genome with a maximum of two mismatches. Finally, according to the downloaded lncRNA genome location information, the probe of the previous step was corresponding to the lncRNA. A total of 4674 probes from the HG-U133_Plus_2 array were uniquely mapped to lncRNAs. In the end, we constructed the lncRNA expression profile include 3724 lncRNAs in these data sets.

### Differential Expression Analysis

Based on the results of the ESTIMATE analysis, we obtained microenvironmental scores in colorectal patients. Subsequently, we classified patients into groups with high or low immune scores and high or low stromal scores based on median scores. Then, differential expression analysis between the high and low groups was performed using the “limma” R package. The differentially expressed lncRNAs (DELs) were identified with an adjusted P-value < 0.05 and an absolute log2 fold change ≥ 0.263.

On the other hand, we also divided the patients into high- and low-risk score groups based on our m6A-TME-LM and analyzed the differentially expressed genes in the groups. The differentially expressed genes (DEGs) were identified with an adjusted P-value < 0.05 and an absolute log2 fold change ≥ 0.585.

### Pearson Analysis Between m6A-Related Genes and DELs

The expression matrix for 21 m6A-related genes was extracted from two separate data sets based on previously published articles. Then, Pearson correlation coefficient (PCC) was used to evaluate the expression correlation between these m6A-related genes and DELs. LncRNAs with PCC ≥ 0.3 or PCC ≤ −0.3 and P-value < 0.05 were selected as m6A-related lncRNAs. Only lncRNAs that meet the threshold in the two data sets will be selected for subsequent analysis.

### Regression Analysis

The m6A-associated differential lncRNAs filtered by two data sets were intersected to get 25 common m6A-associated lncRNAs. Subsequently, univariate cox regression analysis was used to screen out the prognostic lncRNAs. Next, LASSO Cox regression was performed using the R package “glmnet” (23) to construct a tumor microenvironment and m6A-associated lncRNA prognostic model (m6A-TME-LM) of CC patients, which included four m6A-associated lncRNAs.

### Survival Analysis

Kaplan-Meier analysis was used to screen vital prognostic lncRNAs. Survival curves were drawn to illustrate the associations of expression levels of these lncRNAs with CC outcome by the “survival” R package. The multivariable Cox regression analysis was used to test whether the signature was independent of other clinical features. Using pROC package, the area under the ROC curve (AUC) was calculated to assess the prognostic efficiency of this signature.

The survival analysis was also used to compare OS between different subgroups, including low-risk and high-risk subgroups, based on the expression of four m6A-associated lncRNAs. The subgroups were separated by the following features: chemotherapy adjuvant (yes or no), gender (male or female), TNM stage (I and II or III and IV), and age (≤ 68 or > 68 years).

### Construction of the Co-Expression Network

Based on the m6A-TME-LM risk score, we separated the CC patients into low- and high-risk groups. Next, differentially expressed genes (DEGs) associated with the four lncRNAs in m6A-TME-LM were screened from mRNA and lncRNA expression profile data using Pearson correlation coefficient (PCC). Those dysregulated lncRNA-mRNA pairs with PCC ≥ 0.3 or PCC ≤ −0.3 and p < 0.05 were selected as co-expressed lncRNA-mRNA pairs and used to construct the co-expression network.

### Function Enrichment Analysis

All the DEGs and the DELs related genes in the study were extracted for further functional enrichment by using the “clusterProfile” R package (24) and Metascape software. Functional enrichment was conducted for the GO terms (25), KEGG pathways (26), and Hallmarks. Functions with a false discovery rate < 0.05 were selected.

## Results

### Identification of Tumor Microenvironment- and m6A-Related lncRNAs in CC Patients

**Figure 2:**
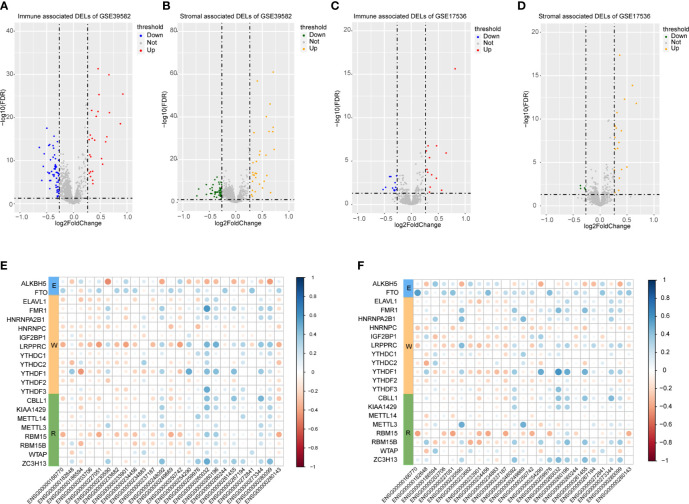
Analysis of microenvironment associated DEGs and Pearson analysis. **(A)** Differential expressed immune associated lncRNAs in GSE39582 data set. **(B)** Differential expressed stromal associated lncRNAs in GSE39582 data set. **(C, D)** Differential expressed immune and stromal associated lncRNAs in GSE17536 data set, respectively. **(E, F)** The correlational heatmap showed coefficient between the common 25 crucial lncRNAs and m6A regulators.

A total of 3724 lncRNAs’ expression levels were re-annotated in two CC data sets. After the ESTIMATE analysis, we divided the CC patients into high- and low-score groups based on the immune or stromal score. Then, we used differential expression analysis to get DELs associated with tumor microenvironments. In GSE39582 data set, there were 86 immune-associated DELs, and 78 lncRNAs were differentially expressed according to immune or stromal score **(** **Figures 2A, B** **)**. Similarly, in GSE17536 data set, there were 25 immune-associated DELs and 23 stromal associated DELs **(** **Figures 2C, D** **)**. Then, we obtained the expression levels of 21 m6A-associated genes through the two data sets separately and performed Pearson correlation analysis between these genes and our m6A-associated lncRNAs in each data set. The lncRNAs with expression values associated with the 21 m6A-associated genes (p < 0.05 and |PCC| > 0.3) were defined as m6A-associated lncRNAs. Finally, we obtained 78 lncRNAs that were significantly associated with m6A in GSE39582 and 36 eligible ones in GSE17536. Finally, 25 m6A-associated lncRNAs were selected in both two data sets **(** **Figures 2E, F** and **Table 1** **)**.

**Table 1:** Basic information of 25 candidate lncRNAs.

ID	Symbol	GSE39582 (N = 585)				GSE17536 (N = 177)			
		Immune Different analysis		Stromal Different analysis		Immune Different analysis		Stromal Different analysis	
		logFC	FDR	logFC	FDR	logFC	FDR	logFC	FDR
**ENSG00000166770**	ZNF667-AS1	0.458	4.18E-22	0.704	1.00E-61			0.455	5.17E-13
**ENSG00000182648**	LINC01006	−0.381	3.33E-14	–	–	−0.273	5.01E-04		
**ENSG00000186594**	MIR22HG	0.602	4.37E-15	0.365	9.46E-06	0.378	3.75E-04	0.399	6.55E-05
**ENSG00000227051**	C14orf132	0.397	1.80E-15	0.646	8.42E-47			0.314	1.83E-11
**ENSG00000230590**	FTX	−0.456	9.46E-08	–	–	−0.314	2.59E-02		
**ENSG00000233901**	RP11-65J3.1	0.351	7.11E-16	0.388	6.67E-20			0.301	1.12E-09
**ENSG00000234883**	MIR155HG	0.672	7.67E-22	0.446	2.70E-09	0.813	2.35E-16	0.346	1.64E-02
**ENSG00000237187**	NR2F1-AS1	0.323	1.27E-11	0.445	4.86E-23			0.279	6.34E-10
**ENSG00000248092**	NNT-AS1	−0.365	3.99E-07	–	–	−0.403	6.13E-04		
**ENSG00000249669**	MIR143HG	0.358	3.01E-08	0.696	7.63E-34			0.276	2.95E-02
**ENSG00000250742**	RP11-834C11.4	0.477	5.01E-26	0.576	7.42E-41			0.350	5.03E-08
**ENSG00000254290**	RP11-150O12.3	−0.497	3.10E-18	−0.396	2.19E-11	−0.347	1.28E-02	−0.365	4.68E-03
**ENSG00000260032**	LINC00657	−0.341	8.74E-09	–	–	−0.380	6.13E-04		
**ENSG00000260196**	RP1-239B22.5	−0.547	2.60E-12	-0.354	1.63E-05	−0.305	2.24E-02		
**ENSG00000260244**	RP11-588K22.2	0.624	2.97E-16	1.158	5.88E-69	0.350	9.92E-03	0.673	1.59E-12
**ENSG00000261455**	LINC01003	−0.324	1.523E-4	–	–	−0.477	1.05E-02		
**ENSG00000267194**	RP1-193H18.2	−0.305	7.711E-4	−0.382	1.18E-05			−0.361	9.98E-03
**ENSG00000272841**	RP3-428L16.2	0.876	2.76E-19	1.463	1.15E-64	0.562	2.29E-02	1.514	1.49E-23
**ENSG00000273344**	PAXIP1-AS1	−0.322	5.39E-08	−0.289	1.62E-06	−0.322	2.13E-02		
**ENSG00000280099**	–	−0.336	3.49E-11	–	–	−0.302	3.04E-03		
**ENSG00000280143**	–	0.319	3.88E-08	0.625	6.16E-34			0.326	2.44E-07
**ENSG00000203706**	SERTAD4-AS1	–	–	0.366	1.92E-14			0.280	2.97E-07
**ENSG00000233682**	RP11-13P5.2	–	–	0.341	2.37E-33			0.275	1.28E-08
**ENSG00000234456**	MAGI2-AS3	–	–	0.405	1.49E-57			0.365	4.40E-18
**ENSG00000259976**	RP11-553L6.5	–	–	0.534	1.25E-10			0.497	3.38E-05

Bold emphasis means P <0.05.

### Identification of Potential Prognostic lncRNAs and Construct the m6A-TME-LM

Combined with clinical information, we screened prognosis-related lncRNAs from the 25 candidate lncRNAs by univariate Cox regression analysis (p < 0.05). We obtained 12 out of the 25 lncRNAs were significantly associated with overall survival (OS) of CC patients **(** **Table 2** **)**.

**Table 2:** The twelve m6A-related prognostic lncRNAs.

Candidate lncRNA	HR	HR.95L	HR.95H	P-value
**ENSG00000237187**	1.653	1.308	2.090	0.001
**ENSG00000234456**	1.943	1.305	2.894	0.001
**ENSG00000233901**	1.564	1.196	2.046	0.001
**ENSG00000254290**	0.724	0.585	0.896	0.003
**ENSG00000166770**	1.379	1.098	1.732	0.006
**ENSG00000227051**	1.360	1.090	1.698	0.007
**ENSG00000250742**	1.366	1.072	1.740	0.012
**ENSG00000267194**	0.838	0.722	0.972	0.020
**ENSG00000249669**	1.212	1.013	1.449	0.035
**ENSG00000233682**	1.576	1.026	2.421	0.038
**ENSG00000182648**	0.776	0.605	0.996	0.046
**ENSG00000260196**	0.856	0.735	0.998	0.047
ENSG00000272841	1.128	0.999	1.275	0.052
ENSG00000203706	1.237	0.975	1.570	0.080
ENSG00000248092	1.147	0.958	1.372	0.135
ENSG00000280143	1.139	0.926	1.400	0.218
ENSG00000273344	0.909	0.731	1.129	0.388
ENSG00000260244	1.071	0.914	1.255	0.398
ENSG00000259976	1.068	0.916	1.245	0.398
ENSG00000280099	1.109	0.868	1.417	0.409
ENSG00000261455	0.952	0.810	1.118	0.547
ENSG00000234883	0.957	0.804	1.139	0.619
ENSG00000230590	1.035	0.893	1.200	0.645
ENSG00000260032	0.960	0.775	1.188	0.706
ENSG00000186594	0.975	0.832	1.143	0.754

Bold emphasis means P <0.05.

**Figure 3:**
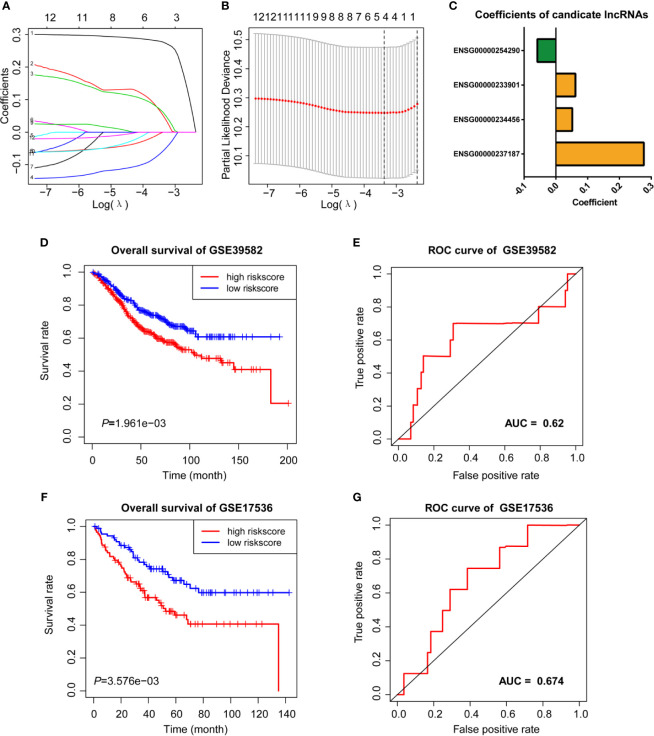
LASSO cox analysis and survival analysis of m6A-TME-LM. **(A–C)** LASSO regression was performed, calculating the minimum criteria **(A, B)** and coefficients **(C)**. **(D)** Kaplan–Meier curves showed that the high-risk subgroup had worse overall survival than the low-risk subgroup in GSE39582 data set. **(E)** Receiver operating characteristic (ROC) curves of m6A-TME-LM for predicting the overall survival in GSE39582 data set. **(F)** Kaplan–Meier curves showing that the high-risk subgroup had worse overall survival than the low-risk subgroup in GSE17536 data set. **(G)** Receiver operating characteristic (ROC) curves of m6A-TME-LM for predicting the overall survival in GSE17536 data set.

We then performed LASSO Cox analysis based on the 12 prognostic lncRNAs in the GSE38592 data set and generated a model which contains four lncRNAs **(** **Figures 3A, B** **)**. The risk score of each sample in the data set was calculated based on the coefficient of four lncRNAs involved in m6A-TME-LM **(** **Figure 3C** **)**. Then, patients were divided into two subgroups based on the median risk score (low-risk and high-risk). The survival curve showed that patients with higher risk scores had a worse prognosis **(** **Figure 3D** **)**. And the ROC curves likewise found that m6A-TME-LM had a good efficacy to predict survival time in this data set. (AUC = 0.63; **Figure 3E** **)**.

### Validation of the m6A-TME-LM

To testify the prognostic efficacy of m6A-TME-LM, we conducted the model in GSE17536 data set using the same algorithm. The low-risk and high-risk subgroups were also distinguished based on the median risk scores of CC patients in this data set. Ultimately, we obtained results consistent with the training data set **(** **Figure 3F** **)**. The ROC curve also indicated that this model had a more vital prognostic ability in the data set (AUC = 0.674; **Figure 3G** **)**. Meanwhile, an independent test data (TCGA data set) was used to testify the predictive efficacy of the model further. We also got consistent results **(** **Supplementary Figure 1A** **)**. The ROC curve also indicated that this model had a stronger prognostic ability in the data set (AUC = 0.986). These results demonstrated the m6A-TME-LM had a stable predictive power of prognosis in CC.

### Survival Analysis of the Four Crucial lncRNAs

**Figure 4:**
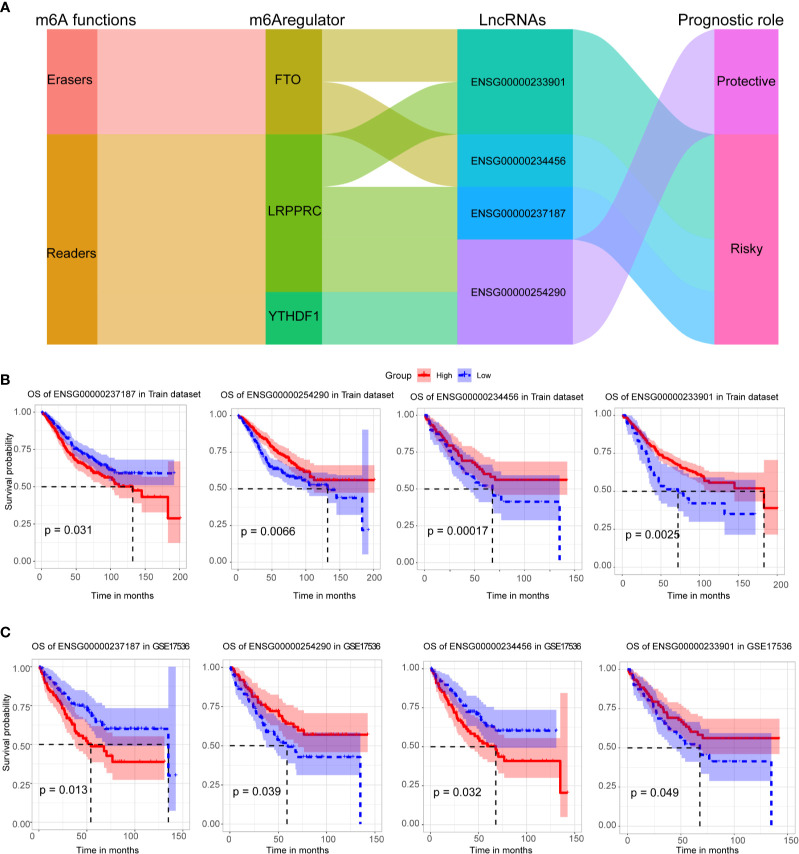
Survival analysis of four crucial lncRNAs. **(A)** The alluvial plot showed the roles of these four lncRNAs in CC. **(B)** Kaplan–Meier curves showed the four lncRNAs’ survival efficacy in GSE39582 data set. **(C)** Kaplan–Meier curves showed the four lncRNAs’ survival efficacy in GSE17536 data set.

Four crucial lncRNAs in the model were evaluated by univariate Cox regression analysis. The forest plot shows that ENSG00000254290 is a protective factor with HR (Hazard ratio) < 1, while ENSG00000237187, ENSG00000234456, and ENSG00000233901 are risk factors with HR> 1 in CC patients **(** **Supplementary Figure 1B** **)**. The alluvial plot showed the roles of these four lncRNAs in CC **(** **Figure 4A** **)**. Then, survival curves showed that all the four lncRNAs are survival-associated in both two data sets **(** **Figures 4B, C** **)**.

#### Stratification Analysis of the m6A-TME-LM

**Figure 5:**
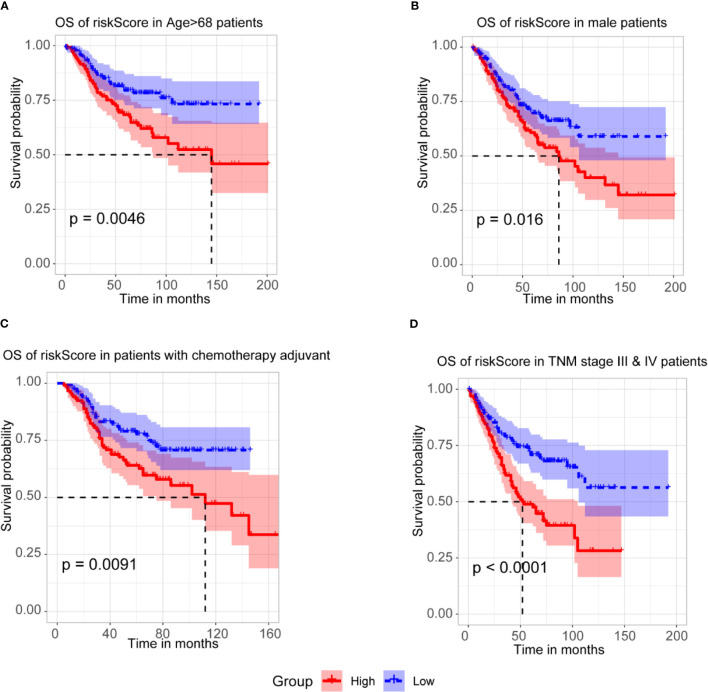
Stratification Analysis of the m6A-TME-LM. **(A)** CC patients with higher risk score of m6A-TME-LM had worse survival rate in age > 68 subgroups.**(B)** CC patients with higher risk score of m6A-TME-LM had worse survival rate in male subgroups. **(C)** CC patients with higher risk score of m6A-TME-LM had worse survival rate in chemotherapy adjuvant subgroups. **(D)** CC patients with higher risk score of m6A-TME-LM had worse survival rate in TNM stage III and IV subgroups.

To further assess the prognostic efficacy of m6A-TME-LM, a stratified analysis was used to explore whether the model still could predict OS in different subgroups. The result showed that the higher risk CC patients had a worse survival rate in age > 68 subgroups **(** **Figure 5A** **)**. Similarly, we confirmed that m6A-TME-LM retained its prognostic efficacy of male patients **(** **Figure 5B** **)** and patients with chemotherapy adjuvant **(** **Figure 5C** **)**. Patients with higher risk also had a worse OS in both the TNM stage III and IV subgroups **(** **Figure 5D** **)**. These data proved that the model could be a novel predictor and performed an excellent prognostic efficacy in CC. The m6A-TME-LM may help to improve the prognosis of CC patients.

### The Potential Function of the m6A-TME-LM

**Figure 6:**
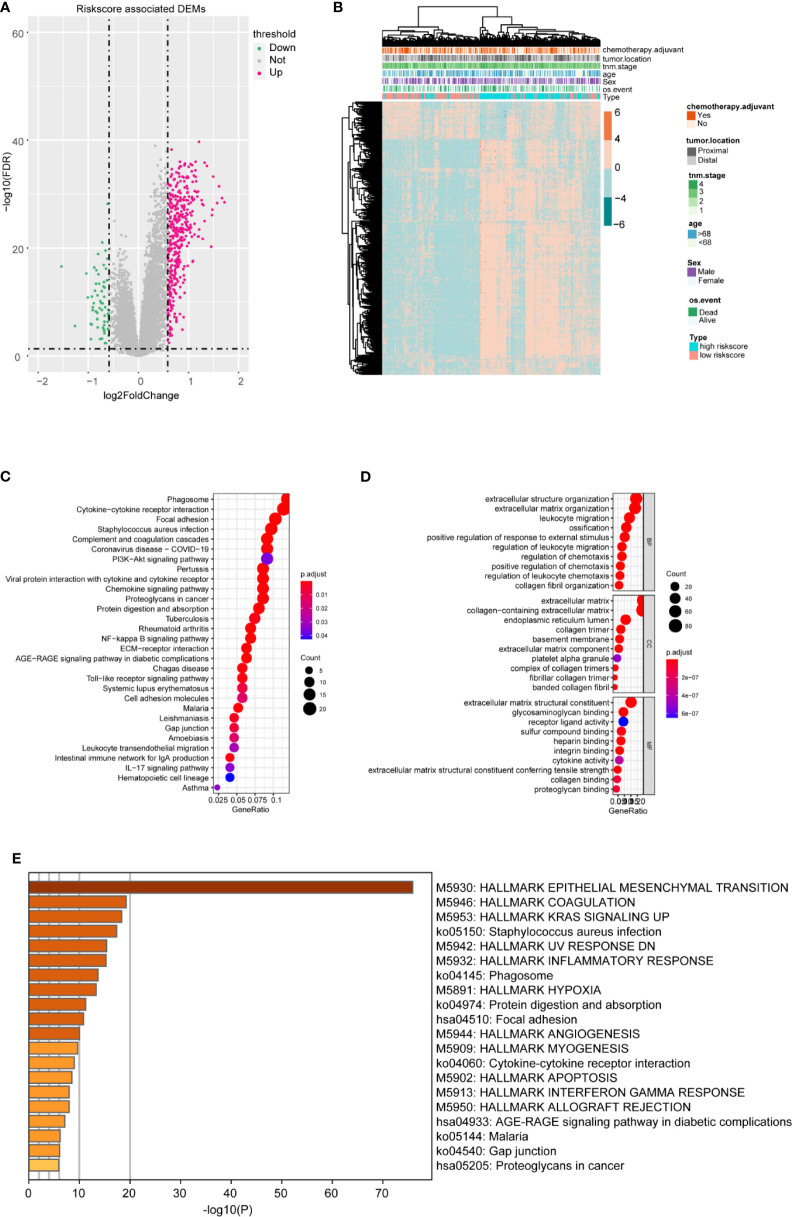
Differential and enrichment analysis of DEGs between high- and low-risk m6A-TME-LM score subgroups. **(A)** Volcano plot showed the differentially expressed genes between the high- and low-risk score groups based on the m6A-TME-LM. **(B)** Heatmap is used for cluster the DEGs expression and clinical traits in CC. **(C)** KEGG enrichment analysis of DEGs. **(D)** Go terms enrichment analysis of DEGs. **(E)** Hallmark function enrichment analysis of DEGs.

To better confirm the signature’s potential functions in CC, we classified the samples into high and low risk‐score groups based on the m6A-TME-LM and obtained DEGs influenced by the model **(** **Figure 6A** **)**. Heatmap showed the cluster efficiency of these DEGs **(** **Figure 6B** **)**. Then, enrichment analysis was performed to confirm the function of the m6A-TME-LM. As shown in **Figure 6C**, the DEGs were enriched in multiple signaling pathways include PI3K-Akt signaling pathway, IL-17 signaling pathway, Toll-like receptor signaling pathway, NF-kappa B signaling pathway, and so on. It also enriched in many GO terms, which contained regulation of leukocyte migration, extracellular matrix, receptor-ligand activity, and so on **(** **Figure 6D** **)**. Further enrichment analysis in Hallmarks showed that these genes involved in the inflammatory response, KRAS signaling and interferon Gamma response, etc. **(** **Figure 6E** **)**. All of these suggest that the m6A-TME-LM plays an essential role in CC.

### Construct the Co-Expressed Network to Explain the Function of the Model

**Figure 7:**
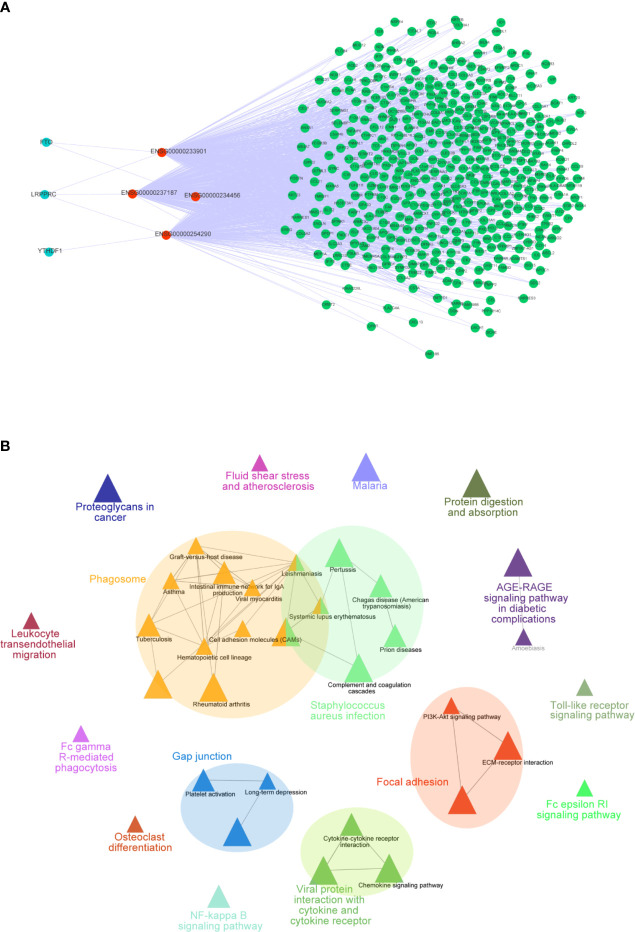
Co-expression network of m6A-TME-LM. **(A)** The network was constructed based on m6A-TME-LM. Red nodes represent lncRNAs, blue nodes represent m6A regulators and green nodes represent mRNAs. **(B)** Enrichment analysis of the co-expression network by ClueGO.

To further explore how these crucial lncRNAs regulate the aberrant expression of mRNAs in CC, we constructed a co-expressed network based on the correlation between the TME-m6A-related lncRNAs and DEGs. Ultimately, four lncRNAs and 906 mRNAs were selected in the network **(** **Figure 7A** **)**. Next, the mRNAs in the network were upload to the ClueGO tool of Cytoscape for functional analysis, and the result showed that these genes were enriched in focal adhesion, AGE-RAGE signaling pathway, Toll-like receptor signaling pathway, and NF-kappa B signaling pathway, etc. **(** **Figure 7B** **)**.

## Discussion

Multiple previous studies have shown that methylation, especially m6A modification, plays a key role as a regulator in cancer development (27), but how it functions in colon cancer progression by regulating lncRNA remains unknown. The m6A regulators have been reported to influence the malignant progression and poor prognosis in a variety of tumors by regulating specific lncRNAs. For example, *METTL3*-mediated m6A methylation modification directly promotes *YAP* translation and increases *YAP* activity by regulating the *MALAT1*-*miR-1914-3p*-*YAP* axis to induce drug resistance and metastasis in NSCLC (28). LncRNA *SOX2OT* elevates *SOX2* expression through *ALKBH5*-mediated epigenetic regulation of glioblastoma and promotes temozolomide resistance (29). The lncRNA *OSER1-AS1* inhibits the growth and metastasis of non-small cell lung cancer by suppressing *ELAVL1* (30). Furthermore, *METTL14*-mediated m6A methylation modifications of *LNC942* promote proliferation and progression in breast cancer cells (31). Numerous other studies have also revealed that m6A modification of lncRNAs can influence cancer development, and lncRNAs can act as co-expression RNAs that target and regulate m6A regulators, thereby affecting tumor invasion progression. In summary, we believe that m6A modification of lncRNAs is not negligible in tumor progression, and we should pay attention to m6A modification of lncRNAs to discover potential cancer prognostic biomarkers.

We identified 25 m6A-related prognostic lncRNAs from 585 CC patients, and four of them were included in the m6A-TME-LM. ENSG00000254290 (*RP11-150O12.3*) was identified as the potential lncRNA biomarkers for liver hepatocellular carcinoma (LIHC) (32). The present study demonstrated that ENSG00000237187 (*NR2F1-AS1*) promoted the progression of NB through the *miR-493/TRIM2* axis. It also regulates *miR-371a-3p/TOB1* axis to suppress the proliferation of CRC cells (33). ENSG00000233901 (*RP11-65J3.1*) is a novel lncRNA that only puts forward in this study, and the potential function needs further analysis. ENSG00000234456 (*MAGI2-AS3*) has been found to be involved in multiple cancers, including esophageal cancer (34), bladder cancer (35), breast cancer (36), and colon adenocarcinoma (37). Compared to other CC-related lncRNA biomarkers (38, 39), these four lncRNAs are relatively novel and have good prognostic efficacy. Therefore, we hope that our results will be helpful in identifying potential targeting prognostic lncRNAs for m6A regulators, thus providing insights for their deeper study in CC tumorigenesis and progression.

## Conclusion

First, we identified the immune-related DELs by ESTIMATE analysis and obtained several m6A-related lncRNAs by correlation analysis. Then, we developed the m6A-TME-LM by lasso regression. Next, survival analysis was used to find potential prognostic biomarkers, and four lncRNAs with independent prognostic efficacy were obtained. Finally, the co-expression network of these lncRNAs helps us to find the potential functions of the crucial lncRNAs. This study may be helpful for future studies concerning CC with the aim of finding potential prognostic targets of it.

## Data Availability Statement

The original contributions presented in the study are included in the article/**Supplementary Material**. Further inquiries can be directed to the corresponding authors.

## Author Contributions

HZ, LZ, TL, and XD conceived and devised the study. HZ, LZ, and SL performed bioinformatic and statistical analysis. JW and CF found testify data and analysis tools. HZ, TL, and XD supervised research and wrote the manuscript. All authors contributed to the article and approved the submitted version.

## Funding

This work was supported by the National Natural Science Foundation of China (Research Index: 81871317), “Winter Olympic Emergency Medical Support” (Research Index: 2019YFF0302300) sponsored by Ministry of National Science and Technique, and Project (RDE2020-15) supported by Peking University People’s Hospital Scientific Research Development Funds.

## Conflict of Interest

The authors declare that the research was conducted in the absence of any commercial or financial relationships that could be construed as a potential conflict of interest.

## Supplementary Material

Supplementary Figure 1 Validation of the m6A-TME-LM. **(A)** Kaplan–Meier curves showed that the high-risk subgroup had worse overall survival than the low-risk subgroup in test data set (TCGA). Receiver operating characteristic (ROC) curves of m6A-TME-LM for predicting the overall survival in TCGA data set. **(B)** Forest plot showed the result of univariate Cox regression analysis.
