# Supplementary figures and images for: N6-Methylandenosine-Related lncRNAs in Tumor Microenvironment Are Potential Prognostic Biomarkers in Colon Cancer
Source: Front Oncol. 2021 Jun 11;11:697949. doi: 10.3389/fonc.2021.697949 (PMC8231021; doi:10.3389/fonc.2021.697949)

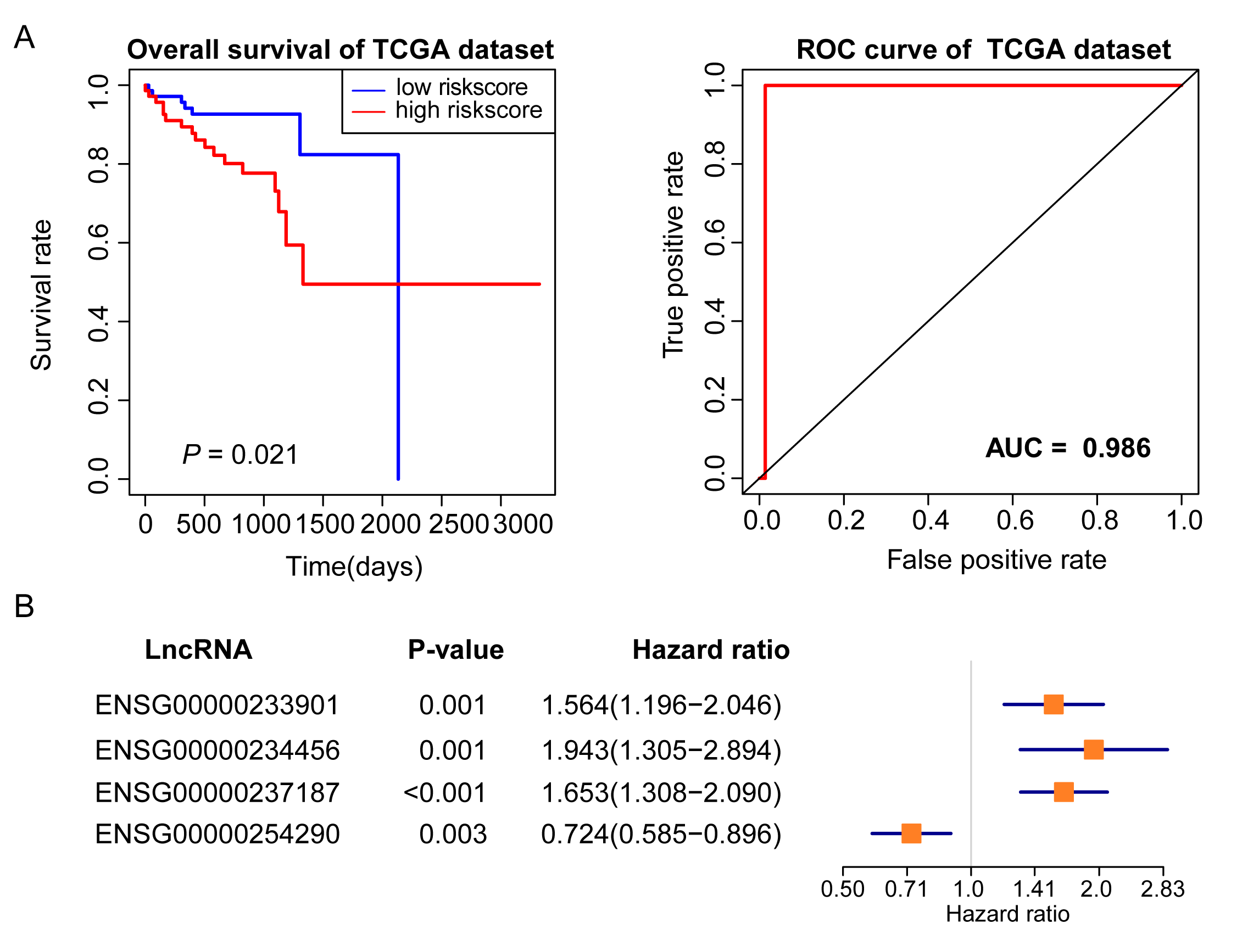

Supplement: Supplementary Figure 1 — Validation of the m6A-TME-LM. (A) Kaplan–Meier curves showed that the high-risk subgroup had worse overall survival than the low-risk subgroup in test data set (TCGA). Receiver operating characteristic (ROC) curves of m6A-TME-LM for predicting the overall survival in TCGA data set. (B) Forest plot showed the result of univariate Cox regression analysis. [file Image_1.tif]
